# Circular RNA DENND1B contributes to cognitive impairment in Alzheimer’s disease by enhancing blood‐brain barrier permeability via transcellular regulatory axis

**DOI:** 10.1002/alz.086245

**Published:** 2025-01-03

**Authors:** Zhi Cheng, Yuling Zhang, Feng Wang

**Affiliations:** ^1^ Tianjin normal University, Tianjin, Tianjin China; ^2^ Tianjin Beichen Traditional Chinese Medicine Hospital, Tianjin, Tianjin China; ^3^ Emory University School of Medicine, Atlanta, GA USA

## Abstract

**Background:**

Circular RNA represents a distinctive form of noncoding RNA resulting from back‐splicing of exons and introns in mRNA. CircRNA has been shown play important roles in neurological diseases, such as Alzheimer’s disease (AD). Some recent studies also have demonstrated circRNA is enriched in the mammal brain and differentially altered during AD. However, little is known the characteristics and functions of key circRNAs directly linked to cognitive of AD. Herein, we identified circDENND1B as a novel circRNA implicated in AD and elucidated its role in cognitive impairment.

**Methods:**

We generated rRNA and linear RNA depleted, and RNA sequencing data from 12‐month‐old 5 × FAD mice hippocampus. The circDENND1B knockdown nanoparticles were applied in 10‐month‐old 5 × FAD mice. The novel object recognition and Morris water maze tests were used to observe cognitive function in these 12‐month‐old mice, and conducting electrophysiological experiments, Golgi‐Cox staining, and measuring blood‐brain barrier permeability improvement also performed in the 5 × FAD mice after circDENND1B knockdown. The fluorescence staining, RNA in situ hybridization, RNA antisense purification, RNA pull‐down, RT‐qPCR, Western blot were quantify the functions of circDENND1B in mice brain and vitro human BBB model.

**Results:**

CircDENND1B, a circRNA enriched in hippocampal neurons, was significantly up‐regulated in AD mice and patients. Knockdown of circDENND1B markedly improved cognitive dysfunctions associated with AD. Additionally we revealed that miR‐17‐5p served as a crucial downstream mediator of circDENND1B‐regulated Blood‐brain barrier (BBB) permeability. Notably, key targets of miR‐17‐5p included lncKCNQ1OT1/VegfA, which involved in the regulation of BBB as well as learning and memory. Overexpression of miR‐17‐5p altered circDENND1B and lncKCNQ1OT1 transcellular shuttling, thereby regulating VegfA expression in brain microvascular endothelial cells leading to changes in BBB permeability.

**Conclusion:**

In conclusion, we demonstrated elevated circDENND1B levels in AD patients and provided evidence that the circDENND1B/lncRNA KCNQ1OT1/miR‐17‐5p/VegfA transcellular regulatory axis plays a role in regulating BBB permeability and neuron inflammatory response in both *in vivo* and *in vitro* AD models. This identified that circDENND1B/lncRNA KCNQ1OT1/miR‐17‐5p/VegfA transcellular regulatory axis may serve as a novel biomarker for AD, offering new insights for prediction, prognosis, and treatment of AD patients.

